# Laboratory Study on Improvement of Expansive Soil by Chemically Induced Calcium Carbonate Precipitation

**DOI:** 10.3390/ma14123372

**Published:** 2021-06-18

**Authors:** Shaoyang Han, Baotian Wang, Marte Gutierrez, Yibo Shan, Yijiang Zhang

**Affiliations:** 1 Key Laboratory of Ministry of Education for Geomechanics and Embankment Engineering, Hohai University, 1 Xikang Rd., Nanjing 210098, China; hanshaoyang0419@163.com (S.H.); btwang@hhu.edu.cn (B.W.); 2 Department of Civil and Environmental Engineering, Colorado School of Mines, 1500 Illinois St., Golden, CO 80401, USA; 3 Geotechnical Engineering Department, Nanjing Hydraulic Research Institute, 34 Hujuguan Rd., Nanjing 210024, China; ybshan@nhri.cn (Y.S.); zhangyijiang1025@163.com (Y.Z.)

**Keywords:** expansive soil, precipitation technique, calcium carbonate, soil improvement

## Abstract

This paper proposes the use of calcium carbonate (CaCO_3_) precipitation induced by the addition of calcium chloride (CaCl_2_) and sodium carbonate (Na_2_CO_3_) solutions as a procedure to stabilize and improve expansive soil. A set of laboratory tests, including the free swell test, unloaded swelling ratio test, unconfined compression test, direct shear test, scanning electron microscopy (SEM) test, cyclic wetting–drying test and laboratory-scale precipitation model test, were performed under various curing periods to evaluate the performance of the CaCO_3_ stabilization. It is concluded from the free swell tests and unloaded swelling ratio tests that the addition of CaCl_2_ and Na_2_CO_3_ can profoundly decrease soil expansion potential. The reduction in expansion parameters is primarily attributed to the strong short-term reactions between clay and stabilizers. In addition, the formed cementation precipitation can decrease the water adsorption capacity of the clay surface and then consequently reduce the expansion potential. The results of unconfined compression tests and direct shear strength tests indicated that the addition of CaCl_2_ and Na_2_CO_3_ has a major effect on geotechnical behavior of expansive soils. Based on the SEM analyses, new cementing crystalline phases formatted by sequentially mixing CaCl_2_ and Na_2_CO_3_ solutions into expansive soil were found to appear in the pore space, which results in a much denser microstructure. A laboratory-scale model test was conducted, and results demonstrate the effectiveness of the CaCO_3_ precipitation technique in stabilizing the expansive soil procedure. The test results indicated that the concentration of CaCl_2_ higher than 22.0% and Na_2_CO_3_ higher than 21.2% are needed to satisfactorily stabilize expansive soil. It is proposed to implement the precipitation technique in the field by the sequential permeation of CaCl_2_ and Na_2_CO_3_ solutions into soils in situ.

## 1. Introduction

Expansive soils are known to exhibit large volume changes due to moisture fluctuations [1,2]. These soils expand due to an increase in water content and shrink due to drying. The swelling and shrinkage of expansive soils often induce large swelling pressures that can cause severe damage to shallow engineering structures such as runways, building foundations, roads and embankments [3,4,5]. In the US alone, it is estimated the expansive soils cause amounting to about US$27 billion in pavement maintenance in 2013 [6]. One of the most effective solutions to mitigate the negative impacts of expansive soils is soil stabilization by treating and improving expansive soils to reduce their swelling and shrinkage potential.

Several methods have been introduced for the treatment of expansive soil [7,8,9]. Chemical stabilization is one of the effective techniques to overcome the undesirable behavior of expansive soils. Among the chemical stabilization methods, traditional treatments such as physical mixing of lime, cement, fibers and fly-ashes with soils have been commonly used for the soil treatment [10,11,12,13,14,15]. However, these traditional methods show some limitations in engineering applications. For example, the addition of lime or cement may have environmental impacts since the production of lime and cement is energy-intensive and emits substantial greenhouse gas into the atmosphere [16,17]. Moreover, the physical mixing of the stabilizers with soils is costly and resource-intensive, making it unsuitable for laboratory-scale applications. In addition, due to high plastic nature of expansive soils, working with and achieving appropriate pulverization in the field are difficult tasks [18]. In-situ stabilization methods using lime column, lime piles and lime slurry injection have been widely used in Japan, China, United States and Sweden. However, being limited by the often low permeability and stiff nature of expansive soil, these in situ methods are ineffective or difficult to implement in some regions [19,20]. Accordingly, in situ stabilization of expansive soil without the need for extensive earthwork is of interest in civil engineering.

Recently, calcium carbonate (CaCO_3_) precipitation has been extensively used to enhance the engineering properties of various materials, such as soil, mortar, concrete and recycled aggregates [21,22,23]. Microbially induced calcite precipitation (MICP) produces carbonate ions from the hydrolysis of urea, which has been investigated by many researchers [24,25]. However, a high concentration of ammonium is generated and accumulated as the by-product during the hydrolysis of urea, which can be harmful to aquatic ecosystems and can pollute the atmosphere [26]. To overcome the limitations of MICP, directly incorporating precipitated calcium carbonate into clay has also been employed to improve the properties of clay [27]. In fact, unlike soft soils, expansive soils have a stiff to very stiff consistency, and consequently, directly physical mixing of calcium carbonate, and clay incur substantial cost in terms of construction equipment and labor. Additionally, numerous researchers have studied chemically induced calcium carbonate precipitation (CCP), which involves CO_2_ exposure or direct injection to hydrated mortar and cemented soils to accelerate the calcium carbonate precipitation on the surface of recycled aggregates and soils [28,29]. In the practical application of CCP, pipes are injected into the ground while controlling the pump pressure in order to supply CO_2_ gas and Ca(OH)_2_ solution in soils. However, injecting Ca(OH)_2_ (irrespective of quantities) is great of harm to plants and soils due to the highly alkaline feature of Ca(OH)_2_ with potential hazards and toxicity [30]. 

The aim of this paper is to present a new method of a mixing-induced precipitation reaction in expansive soils by the successive permeation of calcium chloride (CaCl_2_) and sodium carbonate (Na_2_CO_3_) solutions into expansive soil according to the following reaction:

(1) $${\mathrm{CaCl}}_{2}+{\mathrm{Na}}_{2}{\mathrm{CO}}_{3}\to {\mathrm{CaCO}}_{3}↓+2\mathrm{NaCl}$$

The resulting calcium carbonate (CaCO_3_) precipitation is expected to improve the properties of expansive soil by flocculation–agglomeration, densification and cementation effects [16,31,32,33]. This process is simply based on the reaction between calcium ions (Ca^2+^) and carbonate ions (CO_3_^2−^). A calcite crystal is usually obtained by this reaction, which is the most stable phase of calcium carbonate form at room temperature and normal atmospheric conditions [34,35]. 

The proposed CaCO_3_ precipitation technique will be far superior to the traditional lime pile technique or the CCP method due to the very high diffusion rates of the solutions in soils. In practical applications, in situ stabilization of expansive soils can be conducted by the successive permeation of CaCl_2_ and Na_2_CO_3_ solutions through the pipes in expansive soils. Moreover, the two solutions can be spread on the surface of the ground and allowed to seep naturally into the soils to stabilize them in situ. Moreover, Na_2_CO_3_ is an important ingredient in many household products, especially in cleaning and disinfecting products. Only in very concentrated solution or in solid form is Na_2_CO_3_ potentially harmful [36]. In addition, CaCl_2_ is extensively introduced into naturally contained soluble carbonate formations for in situ restoration of contaminated soils and groundwater [37]. As the precipitate forms, substantial quantities of heavy metal ions coprecipitate with the calcium carbonate precipitate, and the coprecipitates are insoluble in formation fluids, including groundwater. Compared with the MICP method, the proposed technique is relatively not toxic to humans and vegetation and does not pollute the atmosphere. Therefore, this method can be utilized without posing safety and health problems to humans and relatively minimize the impact on the environment.

A comparative study was conducted by sequential mixing of CaCl_2_ and Na_2_CO_3_ solutions with expansive soil in different concentrations. A series of macro- and microlevel tests, including a free swell test, unloaded swelling ratio test, unconfined compression test, direct shear strength test, scanning electron microscopy (SEM), cyclic wetting–drying test and laboratory-scale precipitation model test, were performed to evaluate the performance and efficiency of the proposed soil stabilization technique. 

## 2. Materials and Methods

### 2.1. Test Materials

Expansive soils from Gaochun District, Nanjing City, China, were used in this investigation. The soil was air-dried, and two separate test batches were obtained by passing soil samples through 0.5 and 2 mm sieves. The 0.5 mm sieved soil was used for the free swell test, and the 2 mm sieved soil was used for determining the standard Proctor compaction, unloaded swelling ratio test, direct shear test and unconfined compression test. The physical and engineering properties of the soil used in the study were determined according to Chinese Standard GB/T 50123-1999 [38] and are presented in Table 1. The data for the untreated natural soils include soil index properties, Atterberg limits, shear strength parameters and unconfined compressive strength. Commercial-grade CaCl_2_ and Na_2_CO_3_ were used as stabilizers for the stabilization of the expansive soil.

**Figure 1:**
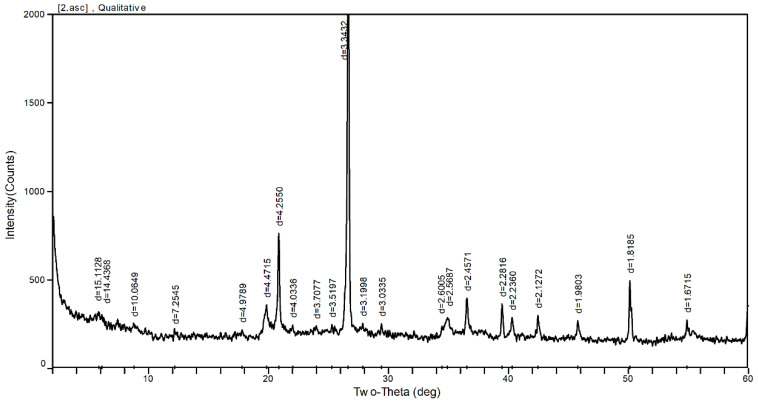
XRD pattern of the expansive soil.

The mineral composition of the expansive soils was characterized by the X-Ray Powder Diffraction (XRD) test. Figure 1 and Table 2 show the XRD pattern and quantitative percentage of the different minerals, respectively, which corresponds to the different crystalline phases, among which montmorillonite was the dominant mineral. The chemical components of the expansive soils were obtained by performing Scanning Electron Microscopy/Energy Dispersive X-Ray Spectroscopy (SEM/EDS, Nanjing Forestry University, Nanjing, China). The chemical elements of the expansive soil are shown in Table 3. Elements Si and Al were characterized as the dominant elements in the expansive soils. 

### 2.2. Sample Preparation

As stated, CaCO_3_ precipitation can be promoted by a double-exchange reaction between CaCl_2_ and Na_2_CO_3_ solutions in expansive soil. According to the reaction in Equation (1), 1 mol of CaCl_2_ (110 g) reacts with 1 mol of Na_2_CO_3_ (106 g) to produce 1 mol of CaCO_3_ (100 g); thus, the molar mass of CaCl_2_ and Na_2_CO_3_ combining to form CaCO_3_ is in the ratio of 1.04 (110/106). Maintaining the same ratio, CaCl_2_ solution and Na_2_CO_3_ solution were mixed sequentially with the expansive soil to precipitate CaCO_3_ in the soil. 

**Figure 2:**
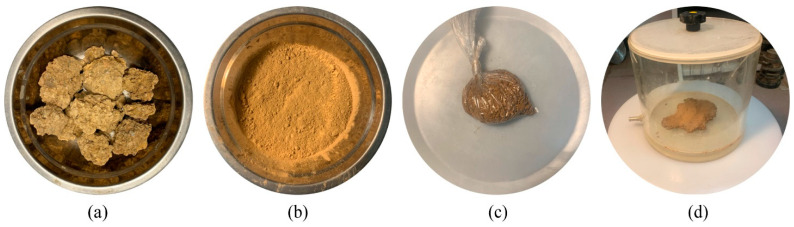
The test procedure: (**a**) initial soil blocks, (**b**) soil after sieving, (**c**) moisture equilibration for 1 h and (**d**) moisture equilibration for 24 h.

Prior to mixing, all the expansive soils were oven-dried and passed through a 2-mm sieve. Then, the expansive soils were separately and thoroughly mixed with the two solutions in equal amounts to an optimum moisture content (OMC) of 22.3%. CaCl_2_ and Na_2_CO_3_ solutions were prepared by dissolving solid CaCl_2_ and solid NaOH, respectively, in water to each achieve 50% concentration. Subsequently, the prepared CaCl_2_ solution (or NaCO_3_ solution) was homogeneously mixed to the needed amount of air-dried soils until they achieved a uniform color. The samples were then placed in an airtight bag for moisture equilibration for 1 h. Subsequently, the prepared Na_2_CO_3_ solution (or CaCl_2_ solution) was mixed manually with the soil samples until the mixture achieved a homogeneous and uniform appearance then placed in an airtight container for moisture equilibration for 24 h. The main steps in the test procedure are shown in Figure 2. 

Thyagaraj et al. [39] found that the sequence of mixing solutions can affect the utility of stabilization; therefore, the expansive soil samples in these tests were mixed with CaCl_2_ and Na_2_CO_3_ in two different sequences. The first sequence is mixing CaCl_2_ solution in expansive soil followed by Na_2_CO_3_, which is called Series 1, while in Series 2, Na_2_CO_3_ solution was mixed before CaCl_2_ solution.

To reveal the effect of excessive CaCl_2_ on the soil properties, two additional mixing methods were designed and tested: (1) Series 1A and Series 2A where the same reaction ratio is maintained while gradually increasing the concentrations of CaCl_2_ and Na_2_CO_3_ solutions to raise CaCO_3_ precipitation in the soil, and (2) Series 1B and Series 2B where the CaCO_3_ precipitation is kept constant while mixing excessive CaCl_2_ in the expansive soil. Table 4 gives the designations and details of the mixing method.

### 2.3. Testing Procedures

It has been known that the stabilization of expansive soils can not only eliminate the swelling potential of soils but also improve their mechanical properties that are of interest in civil engineering applications [40,41]. Therefore, the free swell test and unloaded swelling ratio test were carried out to evaluate the effectiveness of the stabilizers in eliminating swell potential ability. In addition, the direct shear test and unconfined compression test were used to determine the utility of the stabilizers on improving the geotechnical behavior of expansive soils. To further investigate the mechanisms of the soil stabilization process, the microstructure of the natural and treated soil samples was monitored using SEM. In addition, to verify the effectiveness of the CaCO_3_ precipitation technique for stabilizing expansive soil in situ, CaCl_2_ and Na_2_CO_3_ solutions were sequentially permeated into the compacted expansive soil through a central vertical hole, then representative soil samples were collected after 28 days for evaluating the efficiency of in situ CaCO_3_ precipitation technique by performing the free swell tests, unloaded swelling tests, direct shear tests and unconfined compression tests. All the testing procedures used in this paper follow the Chinese Standard GB/T 50123-1999 [38].

The free swell ratio is a simple experimental index to identify the expansion potential of soils [42]. For performing free swell tests, 10 mL of representative oven-dried soil (passing a 0.5 mm sieve) was slowly poured into a 50 mL measuring jar filled with 45 mL distilled water and 5 mL sodium chloride. The soils were then allowed to reach a volume equilibrium state without any change in the volume of the solids in 24 h. Then the final volume of the soils was recorded. The free swell ratio is defined as the increase in the volume of the soil expressed as a percentage of the initial volume of the soil.

Apart from the free swell ratio assessment, the swelling behavior of compacted expansive soil samples is usually identified as an important swelling characteristic that is required for evaluation. An unloaded swelling ratio test was conducted to investigate the compression behavior of the natural and treated soils. The wet homogenized natural and treated soils were compacted in oedometer rings of 60 mm diameter and 20 mm height to the maximum dry density of 1.55 g/cm^3^ using a static press with a piston and then cured with a controlled condition for 1, 7 and 28 days. After curing, the samples were placed between two porous stones with filter papers and set up in an oedometer assembly, and then a dial gauge was installed to measure vertical displacement. A seating load of 1 kPa was applied to make the sample contact with the upper and lower parts of the oedometer assembly, and then the sample was immersed with distilled water for swell potential determination. Dial gauge readings were taken every 2 h until the difference between the two readings is smaller than 0.01 mm. The unloaded swelling ratio is expressed as the ratio of an increased amount of the sample height to initial height after water immersion under no-load conditions.

To investigate the effect of the stabilizers on the unconfined compressive strength of expansive soils, a series of unconfined compression tests were performed. The samples were compacted in molds with 39.1 mm diameter and 80 mm height to the maximum dry density of 1.55 g/cm^3^. To ensure uniform compaction, each sample was evenly divided into five layers of compaction. After each curing period (i.e., 1, 7 and 28 days), the samples were extruded from the molds, and the loading tests were performed.

Shear strength parameters (i.e., cohesion and friction angle) are the principal engineering properties of soil that control the stability of a soil mass under load [43]. To ensure the stability of the constructed facilities on expansive soils, the shear strength parameters of the stabilized compacted soil are evaluated using the direct shear test. The samples for the direct shear test were prepared in a way similar to that used for unloaded swelling tests. Vertical stresses of 50, 100, 150 and 200 kPa were applied and were kept constant as the soil samples were sheared under constant horizontal shear displacement with a velocity of 0.8 mm/min until the horizontal deformation reached the set limit of 4 mm.

To evaluate the micromechanisms of the soil stabilization process by induced CaCO_3_ precipitation, the soil samples were studied and characterized using scanning electron microscopy (SEM). Oven-dried soil samples from direct shear tests were used for SEM analyses. Images of the soil samples were magnified 600, 1200 and 5000 times, respectively. 

The significant damage caused by expansive clay to engineering structures is primarily due to cyclical swell-shrink behavior [44,45]. Therefore, a series of studies were performed to determine the influence of cyclic wetting and drying on the swelling and cracking behavior of natural and treated soil samples. The samples for cyclic wetting–drying tests were prepared similarly to the unloaded swelling test. Apart from determining the influence of cyclic wetting and drying on swelling and cracking behavior of natural and treated soil samples after curing for 7 days, the effects of cyclic wetting–drying on shear strength are also discussed. The samples were performed in conventional oedometer test assemblies with a free surface load. They were allowed to swell upon wetting, and the changes in the thickness of the samples were monitored by using dial gauges. The final thickness upon swell equilibrium was measured, and then the sample was gently removed and placed in a ventilated container, the samples were allowed to shrink, and the masses of the samples were continuously monitored by a balance until the initial mass was reached.

**Figure 3:**
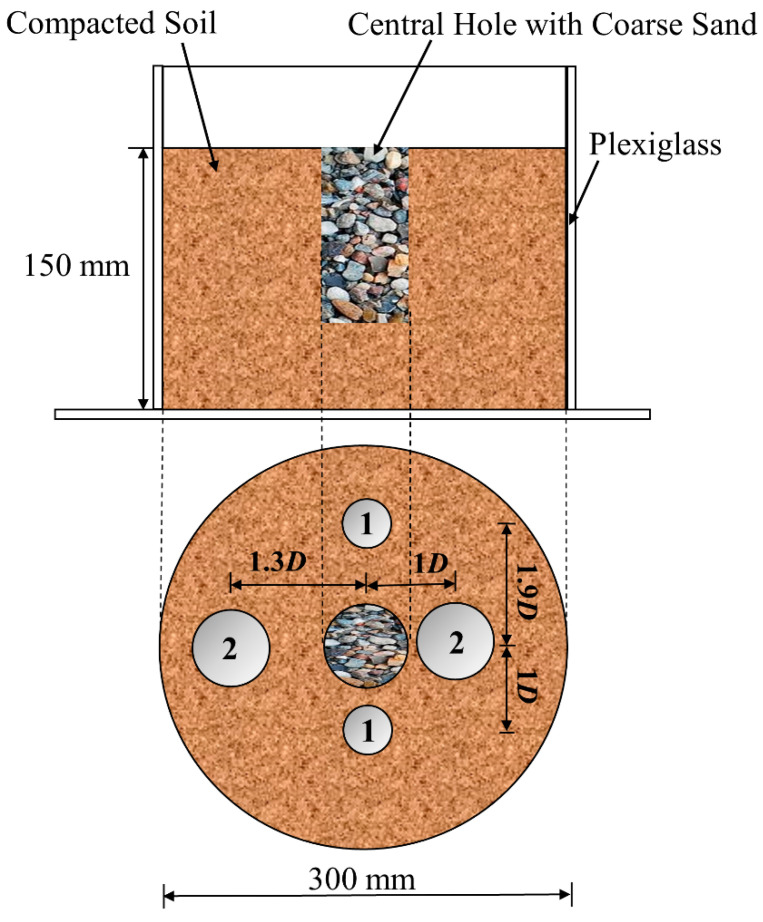
The laboratory-scale model test mold (*D*: diameter of central hole, 70 mm).

The laboratory-scale model test procedure was conducted according to Thyagaraj et al. [46]. The portion of the natural expansive soil passing the 2-mm sieve was divided into 6 equal parts and thoroughly mixed with the desired amount of distilled water to reach the maximum water content of 22.3%. The 6 parts of moist soils were placed to equilibrate in airtight containers for a minimum period of 24 h. At the end of the equilibration period, the 6 parts of soils were thoroughly mixed prior to compaction. The laboratory-scale precipitation model test was conducted in a plexiglass cylindrical mold of 300 mm diameter and 200 mm height (Figure 3). The expansive soil was statically compacted to a thickness of 150 mm (each layer was 30 mm) in a cylindrical test mold. The soil was compacted to a maximum dry density of 1.55 g/cm^3^ at its optimum water content of 22.3%. Each test required 20.09 kg of moist expansive soil (*w* = 22.3%) for compacting the soil to a dry density of 1.55 g/cm^3^. A central hole in 70 mm diameter was created in the compacted soil mass from the top to depth of 100 mm. The volume of this hole is 385 cm^3^, and the hole was filled with coarse sand. Then 2000 mL of 27.5% CaCl_2_ solution was permeated into the soil mass through this central hole in approximately 10 days. Subsequently, 2000 mL of 21.2% Na_2_CO_3_ solution was permeated into the soil mass through the central hole in approximately 10 days to facilitate CaCO_3_ precipitation, according to Equation (1). Then the soil mass was covered with a wet cloth and cured for a period of 28 days. After 28 curing days, the thin-wall sampling tubes with a diameter of 39.1 mm and 80 mm height was pushed into the soil mass with the depth of 120 mm at the radial distance of 0.8·*D* and 1.9·*D* (where *D* = diameter of central hole = 70 mm), from the central hole, respectively, which were used for determining the unconfined compressive strength. Representative soil samples were also collected at a radial distance of 0.8·*D* and 1.9·*D* for the determination of the free swell index. The oedometer rings with a diameter of 60 mm and length of 20 mm were pushed into soil mass with the depth of 50 mm at the radial distance of 1·*D* and 1.3·*D* from the central hole, respectively. The oedometer samples were used for evaluating the unloaded swelling ratio and shear strength. Sequentially, a permeate with CaCl_2_ and Na_2_CO_3_ solutions into the compacted expansive soil through the central hole in the mold. The introduction of the permeate into the sample increased the water content from 22.3% to around 25.0% and reduced the dry density from 1.55 g/cm^3^ to 1.51 g/cm^3^. Therefore, the unconfined compression test and oedometer test samples performed on natural soil under compacted conditions correspond to that of laboratory-scale model test samples. The unconfined compressive strength, free swell index, unloaded swelling ratio and shear strength of laboratory-scale model test stabilized samples were evaluated as per the previously described procedures.

## 3. Results and Discussion

### 3.1. Free Swell Tests

The definition of the free swell ratio *δ_ef_* (%) is given as

(2) $$\delta_{ef}=\frac{V_{we}-V_{0}}{V_{0}}\times 100\%$$

where *V_we_* is the final volume of 10 mL oven dried soil, and *V*_0_ is the initial soil volume (10 mL).

Table 5 gives the results of the free swell ratio tests with the different concentrations of solutions in the two test sequences. The calculated percentage of precipitation by dry weight of soil is also presented in Table 5. According to Equation (1), the amount of precipitation in the soil can be calculated. For example, if 11.2 mL of 11% CaCl_2_ reacts with 11.2 mL of 10.6% Na_2_CO_3_, the amount of precipitation is calculated as follows:

(3) $$11.15\times 11\%\times \frac{100}{110}=1.12\;g$$

The percentage of precipitation by the dry weight of soil is calculated as follows:

(4) $$\frac{1.12}{100}\times 100\%=1.12\%$$

As can be seen in Table 5, an increase in the percentage of CaCO_3_ precipitation profoundly decreased the free swell ratio in both series. The significant reduction of free swell ratio is mainly due to the fact that the divalent calcium ions (source from CaCl_2_) can easily substitute the monovalent ions (e.g., Na^+^) in the diffuse double layer (DDL) of the clay particles [47], thereby leading to a strong short-term reaction between clay and stabilizers, allowing for their flocculation [48]. Therefore, at any given percentage of precipitation, the cases with large amounts of CaCl_2_ show better performance because more Ca^2+^ is provided. Taking the calculated percentage of precipitation of 2.23% in Series 1, for example, the free swell ratio is less than 27% for the case with excessive CaCl_2_, whereas the free swell ratio is 38% for the complete reaction case. Moreover, the addition of CaCl_2_ and Na_2_CO_3_ solutions may increase the salinity of pore liquid, which can decrease the repulsive pressure between clay surfaces, causing the soil skeleton to shrink [40] and therefore leading to a decrease in free swell ratio.

According to Chinese Standard GB 50112-2013 [49], stabilizers can be considered effective by reducing the free swell ratio to less than 40%. Therefore, stabilizers with CaCl_2_ concentration higher than 27.5% and Na_2_CO_3_ concentration higher than 21.5% were selected for subsequent experiments. Furthermore, Series 1 shows higher utility on decreasing the value of free swell ratio than Series 2. Consequently, four cases, namely sequential mixing of 22.0% CaCl_2_ and 21.2% Na_2_CO_3_ solutions, 27.5% CaCl_2_ and 26.5% Na_2_CO_3_ solutions, 27.5% CaCl_2_ and 21.2% Na_2_CO_3_ solutions and 33.0% CaCl_2_ and 21.2% Na_2_CO_3_ solutions, were the preferred proposed mixtures for the subsequent experiments.

### 3.2. Unloaded Swelling Ratio Tests

The unloaded swelling ratio *δ_e_* (%) at time *t* is measured using:

(5) $$\delta_{e}=\frac{z_{t}-z_{0}}{h_{0}}\times 100\%$$

where *z_t_* is the dial gauge reading at time *t* (mm), *z*_0_ is the initial dial gauge reading (mm), and *h*_0_ is the initial height before water immersion (20 mm). Table 6 presents the results of the unloaded swelling ratio test with different concentrations of solutions.

**Figure 4:**
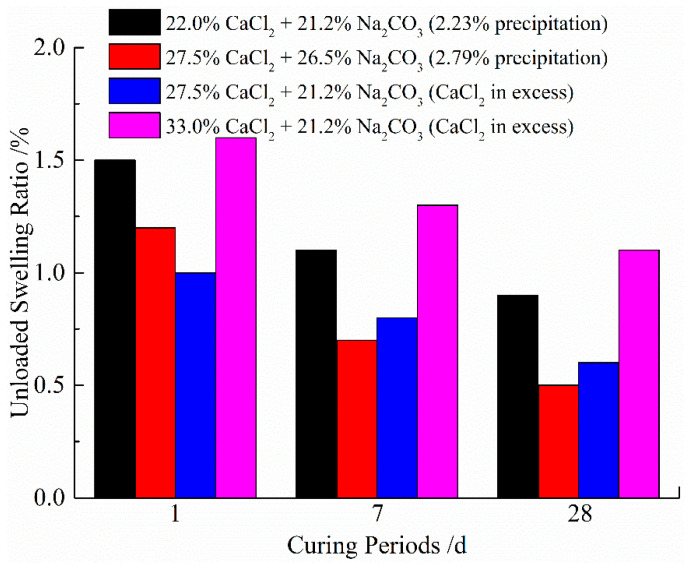
The effect of different concentrations of solutions and curing periods on the unloaded swelling ratio.

Figure 4 shows the effect of different stabilizers on the unloaded swelling ratio for 1, 7 and 28 days of curing time, respectively. Apparently, the unloaded swelling ratio of treated soil samples is all less than 2%, while the unloaded swelling ratio of natural soil is 9.3%. The dramatic reduction in the unloaded swelling ratio by mixing CaCl_2_ and Na_2_CO_3_ solutions can be explained by the diminishment of repulsive forces. The clay particles flocculate by approaching each other to form more compact particle accumulation and aggregation [50,51], and thus, the interaction between the clay surface and water is reduced. Besides, as subsequently discussed, the decrease in the unloaded swelling ratio is also attributed to the formation of new cementation precipitation by solutions reacting by binding the clay particles together and filling the soil voids, causing the decrease in hydration. Moreover, the results in Figure 4 show that the slight decrease in the unloaded swelling ratio with increasing curing period is may be due to the production of new precipitations and the growth of precipitation crystal [52]. 

### 3.3. Unconfined Compression Tests

Table 7 gives the results of the unconfined compression tests with samples prepared by mixing expansive soil with CaCl_2_ and Na_2_CO_3_ solutions in different concentrations. 

**Figure 5:**
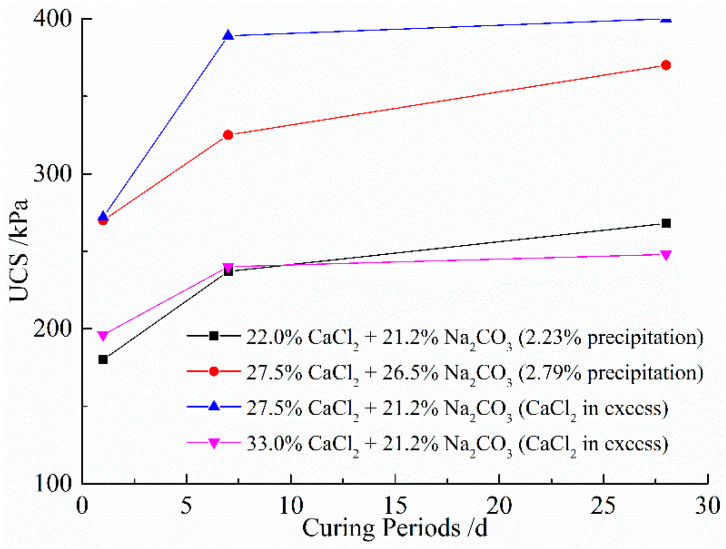
Variations of UCS with different concentrations of solutions versus curing periods.

**Figure 6:**
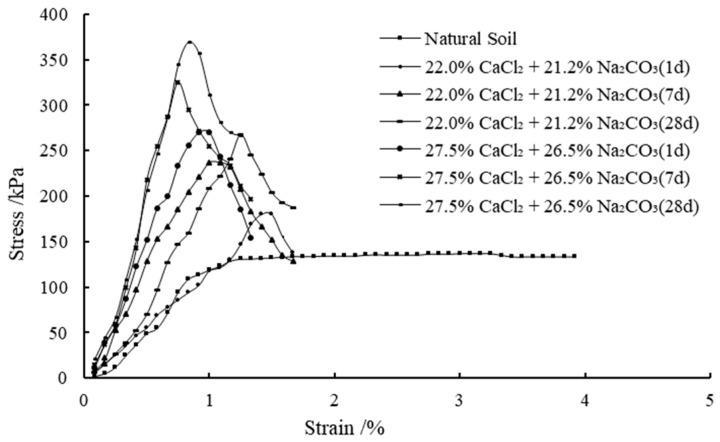
The stress–strain behavior of Series 1 samples with different curing periods.

**Figure 7:**
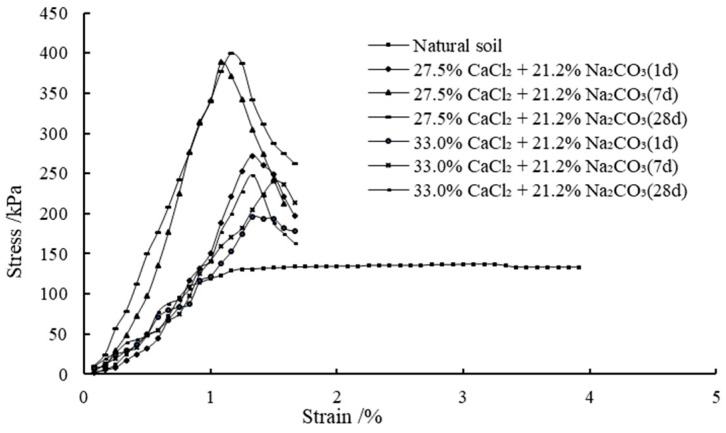
The stress–strain behavior of Series 2 samples with different curing periods.

Figure 5 shows the variations of unconfined compressive strength (UCS) with different concentrations of solutions versus the curing periods of 1–28 days. The stress–strain behavior of all the samples at different curing periods are presented in Figure 6 and Figure 7. It is observed that the treated clay samples show a strengthening trend with the increase in the calculated percentage of precipitations for complete reaction cases. In comparison, the natural soil exhibits UCS of 137 kPa. The significant improvement in the UCS of expansive soil is again primarily attributed to the strong short-term reaction between clay and stabilizers, allowing for flocculation. Besides, according to the reaction in Equation (1), sequential mixing of CaCl_2_ and Na_2_CO_3_ into the expansive soil can form CaCO_3_ precipitation, which will be absorbed on the surface of clay particles and form a package between clay particles, thus generating cementation or bond between clay particles. Moreover, the particle void filled with precipitated CaCO_3_ may increase the soil density in terms of increasing the UCS [28]. A similar stress–strain curve shape is observed in most stabilized cases, which reaches the peak values at lower strains, indicating a significant influence of the stabilization on the material stiffness and overall rigidity. It is interesting to note that the UCS appears to decrease when the concentration of CaCl_2_ is above 27.5% for a constant precipitation content for the curing time of 28 days. Taking the calculated percentage of precipitation of 2.23%, for example, the UCS for the case with 27.5% of CaCl_2_ and 21.2% of Na_2_CO_3_ after 7 days is 389 kPa, whereas the UCS for the case with 33.0% of CaCl_2_ and 21.2% of Na_2_CO_3_ after 7 days is 240 kPa. The reduction in UCS when the concentration of CaCl_2_ is beyond 27.5% may be due to the absorption of more moisture at a higher concentration of CaCl_2_ [53]. As discussed previously, the increase in UCS with the curing period is also contributed by the formation of new precipitations and the growth of precipitation crystals.

### 3.4. Direct Shear Tests

**Figure 8:**
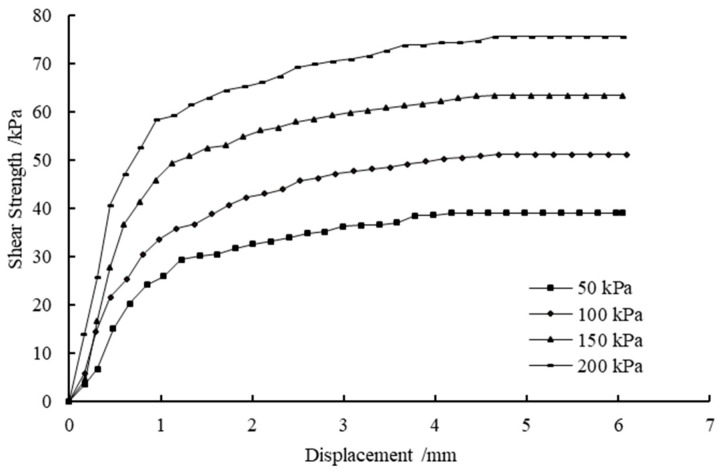
The stress–displacement curve of the soil samples treated with 27.5% CaCl_2_ followed by 26.5% Na_2_CO_3_ solutions.

**Figure 9:**
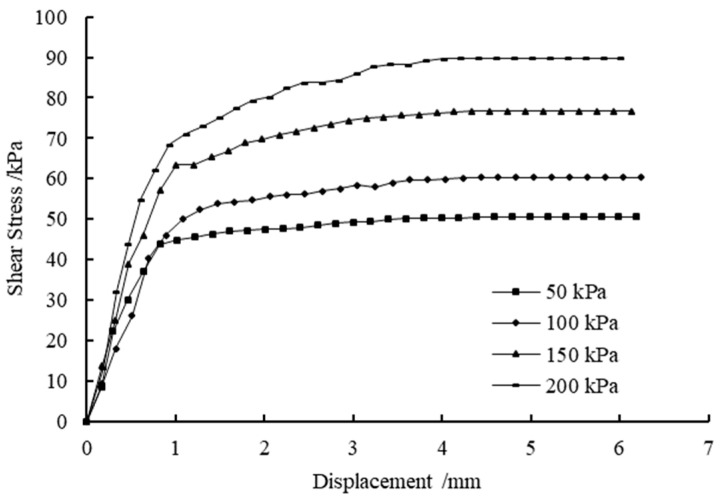
The stress–displacement curve of the soil samples treated with 27.5% CaCl_2_ followed by 21.2% Na_2_CO_3_ solutions.

**Figure 10:**
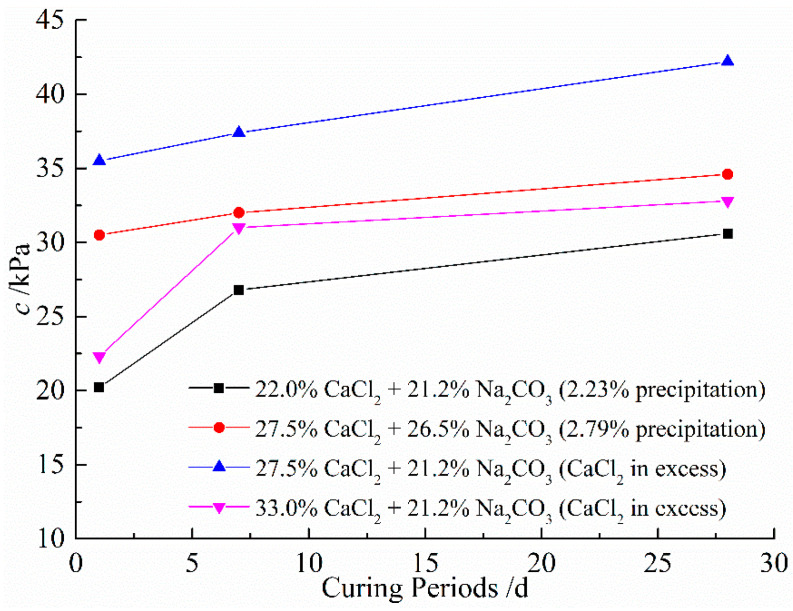
Variations of cohesion with different concentrations of solutions versus curing periods.

**Figure 11:**
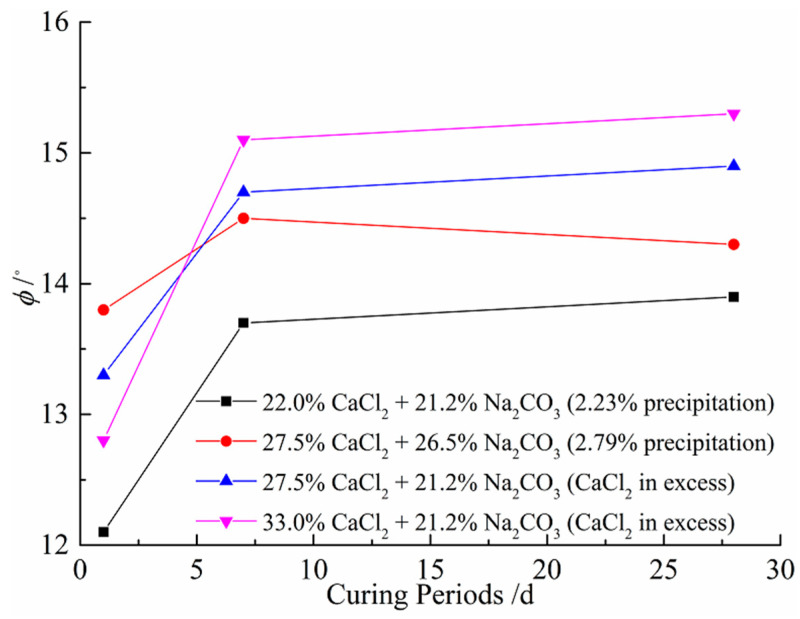
Variations of friction angles with different concentrations of solutions versus curing periods.

Table 8 shows the results of the direct shear tests under different CaCl_2_ and Na_2_CO_3_ concentrations. Figure 8 and Figure 9 show the shear stress-shear displacement curves from the direct shear tests of the soil samples treated with 27.5% CaCl_2_ followed by 26.5% Na_2_CO_3_ solutions and 27.5% CaCl_2_ followed by 21.2% Na_2_CO_3_ solutions, respectively. For reasons of simplicity and clarity, the graphs of the other cases are not presented here as they show the same trends. It can be seen that all curves show similar shapes, and the interface shear stress increases with the increase in shear displacement until it reaches the maximum shear strength, after which it decreases slightly or remains constant. Figure 10 shows the influence of concentrations of solutions versus curing periods on the cohesion of treated soil samples. As illustrated in Figure 10, the increase in CaCO_3_ precipitation results in a significant improvement in the cohesion of the treated samples. This is possibly due to more CaCO_3_ precipitations formed lead to particle–particle cementation and provide a significant improvement in soil properties. On the other hand, the cohesion increases with the concentration of CaCl_2_ until it reaches 27.5%; that is, the cohesion of soil samples will decrease with more addition of CaCl_2_. This phenomenon can be explained by the replacement of native monovalent exchangeable cations by divalent calcium ions, resulting in the loss of double-layer water, which leads to an increase in attraction between soil particles; thus, the effect of preventing the formation of shear slip surface is increased [54,55]. However, excessive CaCl_2_ is more likely to form a face-to-face aggregation or edge-to-edge flocculation, resulting in the gradual increase in the size of the void between particle clusters. Therefore, the cohesive force of clay decreases when the concentration of CaCl_2_ exceeds 27.5%, indicating that a high concentration of CaCl_2_ may have a negative effect on the cohesion of clay [56]. Figure 11 shows the variations in friction angle with concentrations of solutions versus curing periods. As can be seen, the friction angle slightly increases with the calculated percentage of precipitation and concentration of CaCl_2_. This is because the double-layer water is like lubrication between particles, and the strong short-term reaction may decrease the thickness of the diffused double layer, which weakens the lubricating effect and results in the increase in friction angle.

### 3.5. Scanning Electron Microscopy (SEM)

**Figure 12:**
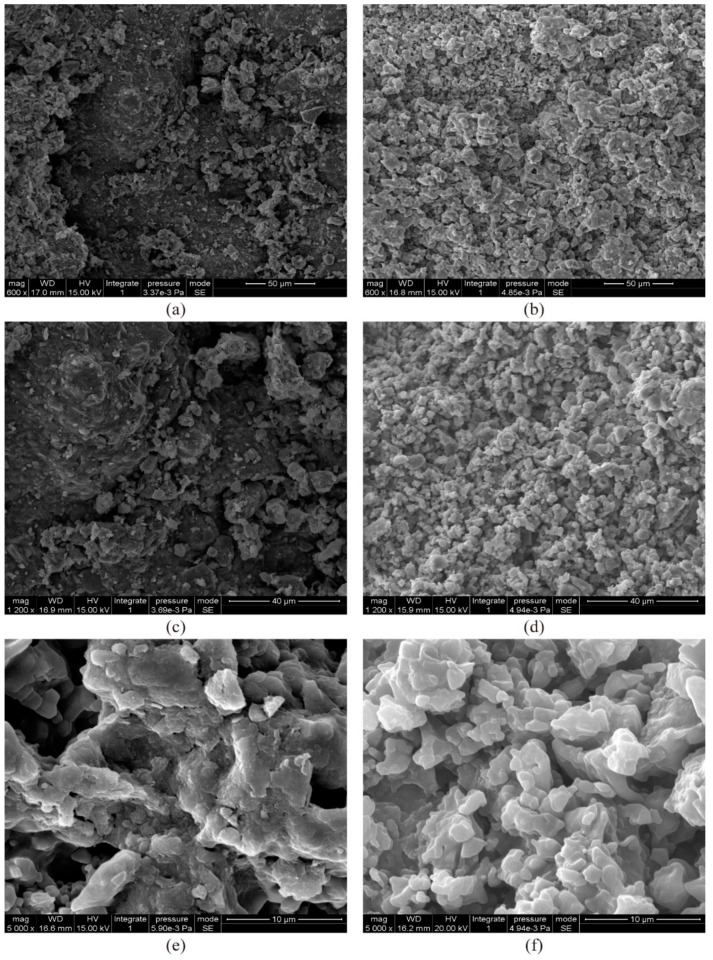
SEM micrographs of: (**a**) natural sample at 600 times magnification; (**b**) treated sample with 27.5% CaCl_2_ and 21.2% Na_2_CO_3_ at 600 times magnification after 7 days; (**c**) natural sample at 1200 times magnification; and (**d**) treated sample with 27.5% CaCl_2_ and 21.2% Na_2_CO_3_ at 1200 magnification after 7 days; (**e**) natural sample at 5000 times magnification; and (**f**) treated sample with 27.5% CaCl_2_ and 21.2% Na_2_CO_3_ at 5000 times magnification after 7 days.

Figure 12a,c,e show the scanning electron micrographs of natural clay samples, while Figure 12b,d,f present the samples treated with 27.5% CaCl_2_ and 21.2% Na_2_CO_3_ solutions after 7 days at 600, 1200 and 5000 times magnification, respectively. As can be seen, the natural clay sample has a dispersed structure and obvious large pore spaces (Figure 12a,c,e). In contrast, the treated sample exhibits more densification and pore-size refinement (Figure 12b,d). The denser microstructure may be due to the precipitations formed in the treated samples (Figure 12f), which distribute spatially within the pore space and bind soil particles together, forming a more integrated composition. The effective cementation and densification of the soil provide significant improvement of soil properties, which is in good agreement with previous works.

### 3.6. Cyclic Wetting–Drying Tests

The wetting–drying process was repeated up to four cycles. The vertical strain *δ_i_* (%) after each cycle is calculated by

(6) $$\delta_{i}=\frac{h_{w}-h_{s}}{h_{s}}\times 100\%$$

where *h_w_* is the thickness of the samples at the end of swelling procedure at cycle *i* = 1, 2, 3, 4; *h_s_* is the thickness of the samples at the end of shrinking procedure at cycle (i−1); when *i* = 1, *h_s_* is the initial thickness of the compacted samples. 

Table 9 presents the results of direct shear tests in terms of cohesion and friction angle after different wetting–drying cycles for compacted expansive soil samples. To simplify the experiment process, the samples after 0, 2 and 4 wetting–drying cycles were used for the shear tests.

**Figure 13:**
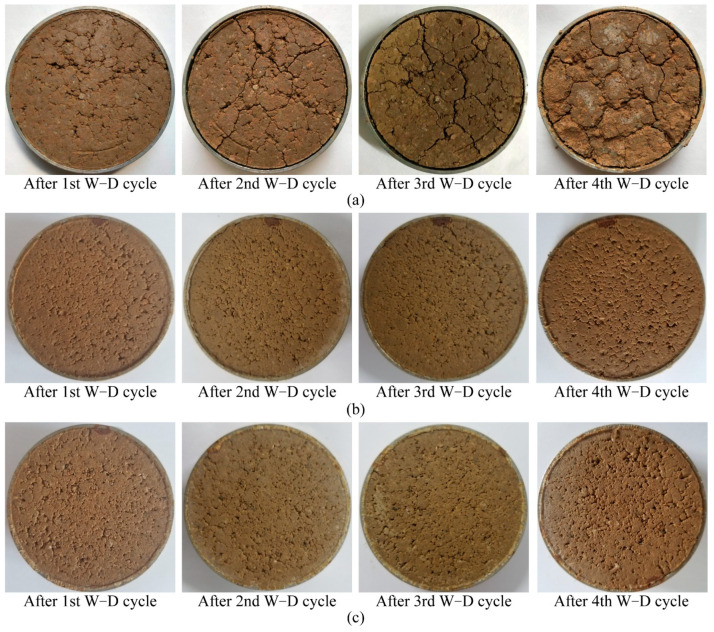
Photographs of surfaces of samples after wetting–drying (W–D) cycles for: (**a**) natural sample; (**b**) treated sample with 27.5% CaCl_2_ and 26.5% Na_2_CO_3_; and (**c**) treated sample with 27.5% CaCl_2_ and 21.2% Na_2_CO_3_.

Figure 13a presents the crack patterns of natural soil samples after each wetting–drying cycle. It can be noted that several microcracks appear on the surface of the natural soil sample after the first cycle, then additional new microcracks were induced by the growing cracks with the increase in wetting–drying cycles. This continuous development and propagation of cracks may form a crack network and weaken the strength of the soil [57]. Figure 13b,c show the soil samples treated with 27.5% CaCl_2_ followed by 26.5% Na_2_CO_3_ solutions and 27.5% CaCl_2_ followed by 21.2% Na_2_CO_3_ solutions, respectively. For reasons of simplicity and clarity, the graphs of the other two cases are not presented here as they show the same results of no cracking. Notably, there are no distinct cracks on the surface of samples after the fourth cycle for both treated soil samples, showing good improvement of the stabilizers.

**Figure 14:**
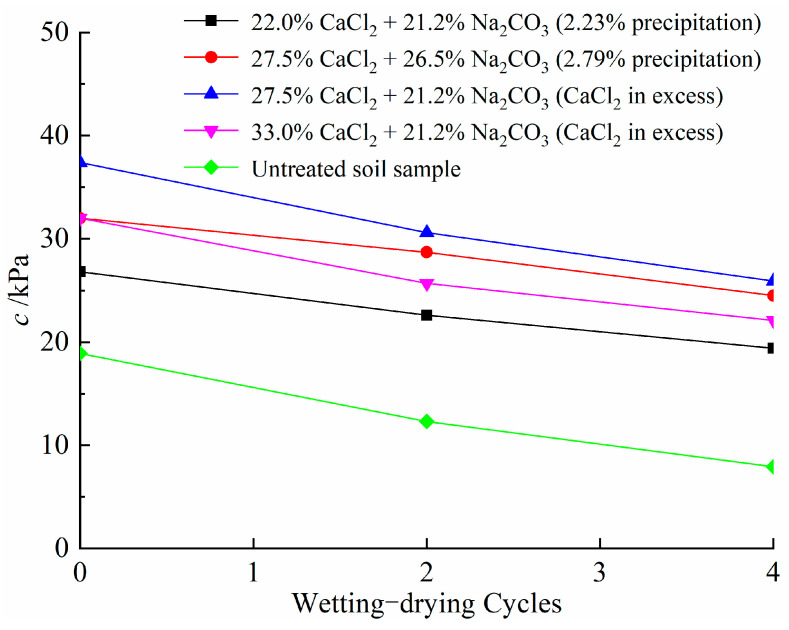
Variations in cohesion versus wetting–drying cycles.

**Figure 15:**
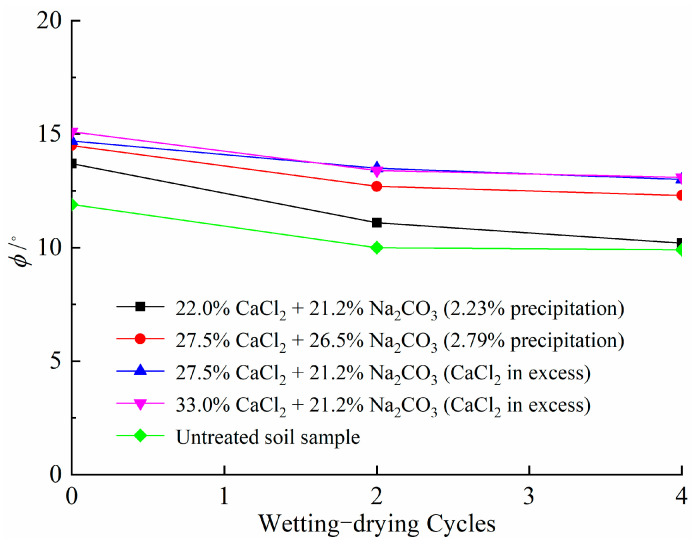
Variations in friction angle versus wetting–drying cycles.

Figure 14 presents the change in cohesion for soil samples after different wetting–drying cycles. Cohesion for all samples decreases with increasing wetting–drying cycles. This can be explained by the successive development of cracks, as shown in Figure 13, which destroys the integrity of soil samples and provides channels for the infiltration of water, resulting in a reduction in cohesion [58]. In comparison, there is still a high value for the cohesion of treated soil samples after experiencing four wetting–drying cycles. This observation is consistent with no distinct cracks appearing on the surface of the samples for both treated soil samples (Figure 13b,c). In addition, the results in Figure 15 show that wetting–drying cycles have a minor effect on the friction angle of soil samples. The moisture content and clay content account for the change in friction angle, as shown by He et al. [59].

**Figure 16:**
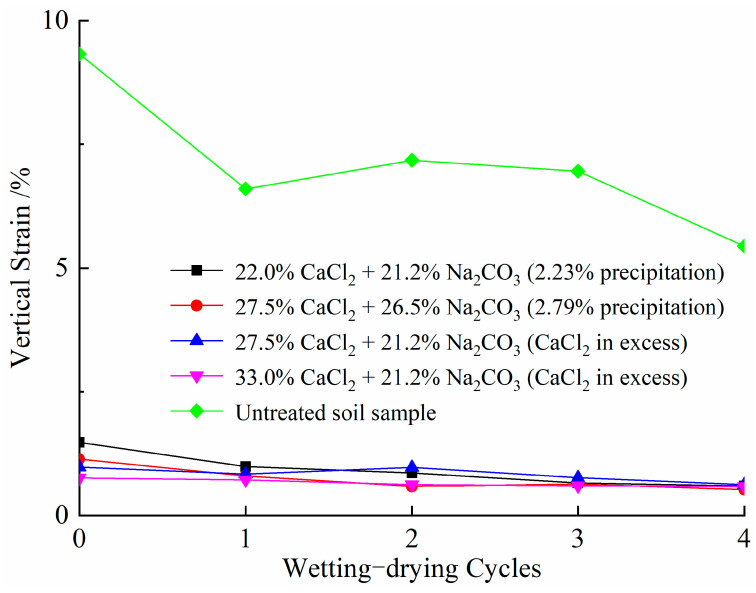
Variations in vertical strain versus wetting–drying cycles for natural and treated samples.

Figure 16 compares the vertical strain calculated from Equation (6) of each cycle calculated versus four wetting–drying cycles for natural and treated samples cured for 7 days. Overall, there is a decrease in the vertical strain with the increase in wetting–drying cycles for natural soil samples. To discuss in detail, an abrupt decrease on the second vertical strain of the natural sample occurs after the first cycle, and the decrease is getting less significant in the later three cycles. This is mainly due to the continuous rearrangement of soil particles and thus leading to the destruction of the internal clay structure [45]. In contrast, both stabilized soil samples show a slight change of vertical strain with wetting–drying cycles, which is attributable to effects of cementation and densification of CaCO_3_ precipitation, as discussed previously. 

### 3.7. Laboratory-Scale Precipitation Model Test

Table 10 compares the results from Tests 1 and 2 samples with those of the natural soil samples. The unconfined compressive strength of Test 1 samples increased from 137 kPa to 270 kPa and 256 kPa at radial distances of 0.8·*D* and 1.9·*D*, respectively. Similarly, the unloaded swelling ratio of Test 2 samples decreased significantly from 9.3% to 0.7–1.0%. The cohesion of the Test 2 samples increased from 18.9% to 28.2–32.4%. The friction angle also slightly increased from 11.9 to 13.0 and 13.3%, respectively. The free swell ratio drastically reduced to 38–39% from 56.0%. The laboratory-scale model test proves the effectiveness of the CaCO_3_ precipitation technique in the in situ stabilization of expansive soil.

## 4. Conclusions

Chemically induced CaCO_3_ precipitation by sequentially introducing and mixing CaCl_2_ and Na_2_CO_3_ solutions into expansive soil was used as a stabilization and soil improvement procedure. Based on the results of free swell tests, unloaded swelling tests, geotechnical strength tests, SEM imaging and wetting–drying tests, the following conclusions can be drawn:

- CaCO_3_ precipitation produced by CaCl_2_ and Na_2_CO_3_ solutions is observed to cause a significant improvement in stabilizing expansive soils. Sequentially mixing CaCl_2_ followed by Na_2_CO_3_ solution shows a better performance than mixing Na_2_CO_3_ followed by CaCl_2_ solution on free swell ratio, decreasing free swell ratio from 32% for the latter case with 21.2% Na_2_CO_3_ and 27.5% CaCl_2_ to 27.0% for the former case with 27.5% CaCl_2_ and 21.2% Na_2_CO_3_, both of which are almost half of the value of 56.0% for natural soil sample.
- Unloaded swelling ratio is decreased from 9.3% for the natural soil sample to 0.6% for the treated sample with 27.5% CaCl_2_ and 21.2% Na_2_CO_3_ after curing periods of 28 days.
- Evident increases in UCS and cohesion are noted with the addition of CaCl_2_ and Na_2_CO_3_ solutions. Furthermore, there is an extra minor increase in the strength of soil samples when the concentration of CaCl_2_ is slightly excessive. However, it is found that there may be a slight decrease in the UCS and cohesion of soil when the concentration of CaCl_2_ is higher than 27.5%. Meanwhile, the concentration of solutions has a minor influence on friction angle.
- According to SEM analysis, the treated sample exhibits more densification and pore-size refinement, presenting denser microstructure than the natural sample.

The proposed chemical improvement method can suppress crack formation effectively during the wetting–drying process, and thus keeping the strength parameters at a relatively high value.

The results of the laboratory-scale precipitation model test show that the precipitation technique significantly reduces the swelling potential and increases the strength properties of the expansive soil. Therefore, the presented laboratory investigation clearly demonstrates the effectiveness of the CaCO_3_ precipitation technique in stabilizing expansive soil. The laboratory-scale model studies also aid in further investigation pilot-scale field trials for evaluating the practical engineering significance. In terms of field implementation of the stabilization procedure, it is also proposed to spray the two solutions on the surface of soils and allow for the solution to seep naturally into the ground to improve expansive soils in situ. This method will not require extensive earthwork and re-working of the soil and is deemed more cost-effective than existing methods.

In further investigation, it is worthy to conduct tests with different concentration types so as to establish a model to predict the strength properties.

## Figures and Tables

**Table 1:** Engineering properties of the expansive soil used in the study.

Property	Value
Specific gravity	2.74
Liquid limit (%)	51.6
Plastic limit (%)	23.2
Plasticity index	28.4
Free swell ratio (%)	56.0
Maximum dry density (g/cm^3^)	1.55
Optimum moisture content ^a^ (%)	22.3
Cohesion ^a^ (kPa)	18.9
Friction angle ^a^ (°)	11.9
Unloaded swelling ratio ^a^ (%)	9.3
Unconfined compressive strength ^a^ (UCS) (kPa)	137

^a^ Sample compacted at optimum moisture content to maximum dry density.

**Table 2:** The results of XRD tests on the expansive soil.

Montmorillonite (%)	Quartz (%)	Vermiculite (%)	Hydromica (%)	Kaolinite (%)	Feldspar (%)	Calcite (%)
25	23	20	15	15	1	1

**Table 3:** Chemical elements of the expansive soil.

Si (%)	Al (%)	Fe (%)	K (%)	Mg (%)	C (%)
41.02	14.26	6.62	3.33	1.08	1.37

**Table 4:** Designations and details of mixing method.

Series Designation			Percentage of Solution Concentration (%)
Natural Soil			-
Series 1: CaCl_2_ followed by Na_2_CO_3_	Series 1A (complete reaction)	Series 1A-1	11.0% CaCl_2_ 10.6% Na_2_CO_3_
		Series 1A-2	22.0% CaCl_2_ 21.2% Na_2_CO_3_
		Series 1A-3	27.5% CaCl_2_ 26.5% Na_2_CO_3_
	Series 1B (CaCl_2_ in excess)	Series 1B-1	27.5% CaCl_2_ 21.2% Na_2_CO_3_
		Series 1B-2	33.0% CaCl_2_ 21.2% Na_2_CO_3_
Series 2: Na_2_CO_3_ followed by CaCl_2_	Series 2A (complete reaction)	Series 2A-1	10.6% Na_2_CO_3_ 11.0% CaCl_2_
		Series 2A-2	21.2% Na_2_CO_3_ 22.0% CaCl_2_
		Series 2A-3	26.5% Na_2_CO_3_ 27.5% CaCl_2_
	Series 2B (CaCl_2_ in excess)	Series 2B-1	21.2% Na_2_CO_3_ 27.5% CaCl_2_
		Series 2B-2	21.2% Na_2_CO_3_ 33.0% CaCl_2_

**Table 5:** The results of free swell tests.

Series Designation	Free Swell Ratio (%)	Calculated Percentage of Precipitation by Dry Weight of Soil (%)
Natural Soil	56	
Series 1A-1	45	1.12
Series 1A-2	38	2.23
Series 1A-3	30	2.79
Series 1B-1	27	2.23
Series 1B-2	24	2.23
Series 2A-1	48	1.12
Series 2A-2	40	2.23
Series 2A-3	35	2.79
Series 2B-1	32	2.23
Series 2B-2	30	2.23

**Table 6:** The results of the unloaded swelling ratio tests.

Series Designation	Curing Periods (d)	Unloaded Swelling Ratio (%)	Calculated Percentage of Precipitation by Dry Weight of Soil (%)
Natural Soil		9.3	
Series 1A-2	1	1.5	2.23
	7	1.1	2.23
	28	0.9	2.23
Series 1A-3	1	1.2	2.79
	7	0.7	2.79
	28	0.5	2.79
Series 1B-1	1	1.0	2.23
	7	0.8	2.23
	28	0.6	2.23
Series 1B-2	1	1.6	2.23
	7	1.3	2.23
	28	1.1	2.23

**Table 7:** The results of unconfined compression tests.

Series Designation	Curing Periods (d)	UCS (kPa)	Calculated Percentage of Precipitation by Dry Weight of Soil (%)
Natural Soil		137	
Series 1A-2	1	180	2.23
	7	237	2.23
	28	268	2.23
Series 1A-3	1	270	2.79
	7	325	2.79
	28	370	2.79
Series 1B-1	1	272	2.23
	7	389	2.23
	28	400	2.23
Series 1B-2	1	196	2.23
	7	240	2.23
	28	248	2.23

**Table 8:** The results of direct shear tests.

Series Designation	Curing Periods (d)	Cohesion (kPa)	Friction Angle (°)	Calculated Percentage of Precipitation by Dry Weight of Soil (%)
Natural Soil		18.9	11.9	
Series 1A-2	1	20.2	12.1	2.23
	7	26.8	13.7	2.23
	28	30.6	13.9	2.23
Series 1A-3	1	30.5	13.8	2.79
	7	32.0	14.5	2.79
	28	34.6	14.3	2.79
Series 1B-1	1	35.5	13.3	2.23
	7	37.4	14.7	2.23
	28	42.2	14.9	2.23
Series 1B-2	1	22.3	12.8	2.23
	7	32.0	15.1	2.23
	28	32.8	15.3	2.23

**Table 9:** Direct shear test results after wetting–drying cycles.

Series Designation	Numbers of Cycles	Cohesion (kPa)	Friction Angle (°)
Natural soil	0	18.9	11.9
	2	12.3	10.0
	4	7.9	9.9
Series 1A-2	0	26.8	13.7
	2	22.6	11.1
	4	19.4	10.2
Series 1A-3	0	32.0	14.5
	2	28.7	12.7
	4	24.5	12.3
Series 1B-1	0	37.4	14.7
	2	30.6	13.5
	4	25.9	13.0
Series 1B-2	0	32.0	15.1
	2	25.7	13.4
	4	22.1	13.1

**Table 10:** Comparison of physicochemical and geotechnical behavior of tests 1 and 2 with natural samples.

Sample	Radial Distance	Unconfined Compressive Strength (UCS) (kPa)	Unloaded Swelling Ratio (%)	Cohesion (kPa)	Friction Angle (°)	Free Swell Ratio (%)
Natural Soil	-	137	9.3	18.9	11.9	56.0
Test 1	0.8D	270	-	-	-	38.0
Test 1	1.9D	256	-	-	-	40.0
Test 2	1.0D	-	1.0	32.4	13.0	-
Test 2	1.3D	-	0.7	28.2	13.3	-

## Data Availability

The data presented in this study are available on request from the corresponding author.
